# Improved EOG Artifact Removal Using Wavelet Enhanced Independent Component Analysis

**DOI:** 10.3390/brainsci9120355

**Published:** 2019-12-04

**Authors:** Mohamed F. Issa, Zoltan Juhasz

**Affiliations:** 1 Department of Electrical Engineering and Information Systems, Faculty of Information Technology, University of Pannonia, Egyetem u.10, 8200 Veszprém, Hungary; juhasz@virt.uni-pannon.hu; 2 Department of Scientific Computing, Faculty of Computers and Informatics, Benha University, Fareed Nada, Benha 13511, Egypt

**Keywords:** EEG, EOG artifacts removal, independent component analysis, discrete wavelet transform (DWT)

## Abstract

Electroencephalography (EEG) signals are frequently contaminated with unwanted electrooculographic (EOG) artifacts. Blinks and eye movements generate large amplitude peaks that corrupt EEG measurements. Independent component analysis (ICA) has been used extensively in manual and automatic methods to remove artifacts. By decomposing the signals into neural and artifactual components and artifact components can be eliminated before signal reconstruction. Unfortunately, removing entire components may result in losing important neural information present in the component and eventually may distort the spectral characteristics of the reconstructed signals. An alternative approach is to correct artifacts within the independent components instead of rejecting the entire component, for which wavelet transform based decomposition methods have been used with good results. An improved, fully automatic wavelet-based component correction method is presented for EOG artifact removal that corrects EOG components selectively, i.e., within EOG activity regions only, leaving other parts of the component untouched. In addition, the method does not rely on reference EOG channels. The results show that the proposed method outperforms other component rejection and wavelet-based EOG removal methods in its accuracy both in the time and the spectral domain. The proposed new method represents an important step towards the development of accurate, reliable and automatic EOG artifact removal methods.

## 1. Introduction

Electroencephalography (EEG) is a non-invasive method for measuring brain activity. Due to its low cost and high temporal resolution, it is routinely used in clinical diagnostics, epilepsy surgery and cognitive psychology research. A major concern when processing EEG measurement data is the presence of various artifacts that are generated by extra-cerebral sources, such as eye blinks and eye movements (electrooculographic/EOG artifacts), muscle movement (neck, jaw and face muscles; electromyogram/EMG artifact) or heart-related EEG disturbances (electrocardiography/ECG artifact and pulse artifact). Unfortunately, these artifacts distort the measured EEG signals and, in the worst case, can make entire measurement datasets unusable. Artifact removal is therefore an essential step in correct EEG data pre-processing [1].

The simplest artifact removal approach is to discard artifact contaminated data segments from the measurement, and process only the remaining clean segments. This approach, however, requires visual data inspection as well as manual rejection of artifact contaminated data segments or epochs. Besides being a very labor-intensive task that requires a trained expert, this method cannot be automated. Furthermore, rejection of data epochs can result in significant loss of trials, which in turn can have adverse effects in event related potential (ERP) studies. Using a reduced number of epochs in averaging can result in critically low signal-to-noise ratio.

More sophisticated artifact removal methods rely on cross-correlation filtering, which, in turn, requires the use of reference signals, obtained using, e.g., horizontal-vertical eye movement (EOG), ECG or electromyography (EMG) sensors. The use of these additional electrodes can be acceptable in strictly controlled laboratory situations, but they can be problematic in clinical settings due to patient discomfort or movement. Hence, much work has been done to develop artifact removal methods that work without external electrodes and can be performed without manual inspection. The most successful such approaches are based on the application of independent component analysis (ICA) [2] that can separate a signal mixture into its original sources (a.k.a. independent components), based on the condition of statistical independence. Assuming that the underlying sources of EOG, ECG and EMG artifacts are independent from other cerebral sources, ICA can separate these artifacts from EEG components. This paper focuses on the automatic removal of EOG artifacts only, using ICA as the underlying method. The traditional approach is to reject an independent component entirely if it contains EOG artifacts. This, however, may lead to loosing important EEG data present in the component [3,4,5]. The wavelet-enhanced ICA method (wICA) proposed by Castellanos and Makarov [6] was shown to reduce information loss and outperform the component rejection-based EOG removal method. The method proposed in this paper improves the wICA method to further reduce neural data loss and signal distortion by performing artifact removal only within the EOG contaminated sections of the ICA components, keeping as much of the relevant EEG information intact as possible. The new method does not require visual inspection and manual intervention, which results in significantly faster pre-processing steps and lays the foundation of high-quality automatic artifact removal.

The structure of the paper is as follows. Section 2 provides an overview of the most influential EOG removal methods. Section 3 describes the proposed new EOG removal method algorithm and its key steps in detail, as well as the performance metrics that will be used in the validation of the method. Section 4 presents the evaluation results obtained using three different types of EEG datasets. The paper ends with discussions and conclusion.

## 2. Related Work

Eye movements and blinks are transient activities that occur relatively infrequently, but unfortunately generate very high amplitude peaks. These artifacts can be easily identified visually in frontal lobe signals waveforms. Since the spectrum of the EOG artifact overlaps the spectrum of the underlying EEG signal, simple filtering methods are unable to remove artifact effects entirely [7]. Adaptive filtering methods based on autoregressive models and reference EOG signals [8] have been used for removing EOG artifacts but these methods do not take into consideration that the reference EOG channels are also contaminated with EEG data, which presents difficulties in obtaining an accurate estimate of the EOG effect [9]. For these reasons, independent component analysis [10] is the method of choice today for EOG artifact removal.

Originally developed for solving the blind source separation (BSS) problem, ICA is a robust method for detecting artifacts by decomposing the EEG signals into their independent source components. Since Makeig et al. [11] suggested the use of ICA for artifact removal, many alternative ICA-based artifact cleaning methods have been proposed. The major differences among the methods are in (i) how artifact components are identified (manual, reference electrode and statistical methods) and (ii) how artifact removal is performed (rejection of entire artifact component and artifact correction within component). Joyce et al. [4] proposed the use of ICA for automatic EOG artifact removal using reference EOG channels for correlation-based artifact component identification followed by full component rejection. The auto-regressive exogenous method (ICA-ARX) [5] also removes ocular artifacts using EOG reference signals. Here input and output signal pairs are required to build the auto-regressive model. A similar approach is used in the popular FASTER [12] and DETECT [13] Matlab-based artifact removal toolboxes. The also widely used ADJUST [14] toolbox, however, does not rely on reference electrodes, it uses spatial and temporal component features to identify EOG components [15]. An example of ICA followed by adaptive filtering based EOG removal is [16].

While ICA-based methods showed encouraging results for EOG artifact removal, it has been also pointed out that ocular sources are not entirely separated from neural sources [4], which makes the full component rejection method a non-ideal solution. Zeroing out the weights of an artifact component before the inverse ICA is performed will also remove all neural data present in that component. To overcome this problem, more sophisticated ICA-based methods were proposed for removing artifacts while retaining the original neural information present in the data.

The discrete wavelet transform (DWT) can be used to decompose a measured signal or its ICA derived independent components into wavelet components using basis functions from wavelet families such as Symlets, Coifs, Haar, etc. [17]. Examples for wavelet-based artifact removal from raw measured data are [15] and [16], in which the wavelet decomposition was combined with statistical approaches to extract artifact features from the decomposed raw EEG signal using the Symlet basis function. Here the assumption is similar to ICA that one wavelet component describes the artifact, which, when removed, removes the artifact from the signal. The most successful approach for artifact removal is the combination of wavelet decomposition and ICA [18,19,20,21]. One approach is to apply ICA to the wavelet decomposed signal components (AWICA) [22]. In the AWICA method, artifacts are detected using statistical measures, such as kurtosis or Renyi’s entropy [23]. The drawback of this approach, however, is that in higher dimensions, Renyi’s entropy incurs high computational cost due to the kernel density needed for the component [24], and the two statistical metrics could not differentiate clearly between EOG and ECG peaks. The same approach was proposed by Kelly et al. [24] where the artifactual coefficients above a threshold were replaced by the median of a set of coefficients outside the artifacts, but they only tested the method on a measured dataset without evaluating the performance on a standard dataset containing EOG artifact annotations.

Another approach is to apply the wavelet transform after ICA decomposition to the artifact independent components (ICs) such as wavelet-enhanced ICA and wICA [6]. In the wICA method, the wavelet transform was used in combination with ICA, relying on the fact that wavelet coefficients of the artifact component typically have higher amplitudes than that of the cerebral activity components, and by zeroing out the coefficients that are larger than a certain threshold, EOG artifacts can be removed from the signal. For successful wavelet-based removal, the threshold selection is crucial. An adaptive threshold method based on DWT was introduced to identify and remove EOG artifacts [25] without losing the related EEG information. However, these methods are not as effective if applied to the raw signal and not ICA components. This approach was modified by Nguyen et al., [26] who introduced the wavelet neural network (WNN; clean and contaminated EEG data is used to train the network) and achieved 9.07 µV root mean square error (RMSE) between the cleaned and the artifact-free data. This method works without a reference EOG signal that is normally required in the linear regression based methods [8]. Burger and van den Heever [3] improved upon this method but their solution can only remove eye blinks; it does not work for eye movement artifacts. Besides wavelet transformation, other decomposition methods have been recommended, such as ensemble empirical mode decomposition for single channel EEG followed by ICA for artifact removal [27].

## 3. Materials and Methods

The goal of the proposed new ICA-based artifact removal method is to keep as much neural information from the original signal as possible. Instead of rejecting an entire EOG independent component, the component is kept but the EOG peaks are first removed from, then the cleaned component is used to reconstruct the EOG-free measurement data.

### 3.1. Independent Component Analysis

Independent component analysis (ICA) was originally developed to solve the blind source separation (BSS) problem [28] and normally refers to a class of algorithms that can recover statistically independent signals (components) from a linear mixture, based on higher-order statistics as a measure of independence. ICA is considered a robust method for identifying and removing artifacts normally found in EEG signals.

A brief formal introduction is as follows. Assume N statistically independent sources, s_i_(t), i = 1, …, N. Suppose that the sources cannot be observed directly, only via N sensors that obtain N observation signals, x(t). The observed signals are mixtures of the original sources. Sensors must be spatially separated (e.g., as the electrodes on the scalp), as each sensor must measure a mixture different from the others. The mixing process than can be described as

(1) $$x_{t}=As_{t},$$

where **A** is the square mixing matrix (spatial weight matrix, channels × components) and **W** = **A**^−1^ is the “unmixing matrix” that must be obtained in order to calculate an estimate ${\hat{S}}_{t}$ of the original sources as

(2) $${\hat{S}}_{t}=Wx_{t}.$$

The following restrictions apply to ICA in order to produce a solution: (i) sources must be statistically independent, (ii) sources cannot have Gaussian distribution and (iii) the mixing matrix must be invertible. The estimation of ${\hat{S}}_{t}$ requires pre-processing steps (dimensionality reduction, centering and uncorrelation). Various ICA variants exist due to differences in the statistical measures used in the separation step [29,30] One of the most popular ICA variants in the EEG community is the Infomax ICA algorithm [31]. Infomax ICA uses a contrast function based on neural network theory and maximizes the output entropy of the neural network. Assuming **x** as the input to the neural network with outputs $\phi_{i}\left(w_{i}^{T}x\right)$, where $\phi_{i}$ is some non-linear function, the goal is to maximize the entropy of the output $L_{2}=H\left(\phi_{1}\left(w_{1}^{T}x\right),\ldots ,\phi_{n}\left(w_{n}^{T}x\right)\right)$ using a stochastic gradient ascend algorithm. For a more detailed description of the theoretical foundations of ICA and ICA algorithm variants, their convergence properties, the quality of source separation or their runtime complexity, the interested reader is referred to the literature [2,3,28,32,33,34,35].

### 3.2. EEG Datasets

Three different sets of EEG measurement data have been selected for the evaluation of our proposed method. These include publicly available datasets as well as data recorded in our laboratory.

Semi-simulated dataset: The publicly available Klados EEG dataset [36] was created for the purpose of EOG artifact removal validations; to serve as a reference dataset that can be used for comparison purposes. Data were recorded from 27 subjects (males and females), using the standard 19 electrode 10–20 layout EEG system, with sampling frequency of 200 Hz, resulting in 54 datasets. Simulated EOG artifacts were then added to the pure, artifact-free data using the following expression:

(3) $${\text{Contaminated\_EEG}}_{i,j}={\text{Pure\_EEG}}_{i,j}+a_{j}V_{EOG}+b_{j}H_{EOG},$$

where ${\text{Pure\_EEG}}_{i,j}$ is the signal obtained with eyes closed (no EOG artifacts), and the $V_{EOG}$ and $H_{EOG}$ terms are the additive vertical and horizontal EOG activities.

PhysioNet EEG datasets: The PhysioNet database contains brain–computer interface datasets [37,38] that were recorded during Brain Computer Interface (BCI) experiments to measure the event-related potential (ERP) of the P300 waves in a spelling experiment. Data were collected using the BioSemi Active Two EEG system, with 64 EEG electrodes and additional vertical and horizontal (VEOG, HEOG) ocular electrodes at 2048 Hz sampling rate.

Laboratory resting-state dataset: We have recorded 2–3 min closed and open eye resting state EEG in our laboratory from 22 adult volunteers (males, age from 16 to 21 years). During the experiment, subjects had to sit and relax in a silent room. Data were recorded using a Biosemi ActiveTwo EEG system (*f_s_* = 2048 Hz) using 128 active electrodes arranged in the ABC radial electrode layout. The volunteers gave their written consent for participating in the experiments.

### 3.3. EOG Artifact Removal Algorithm

**Figure 1:**
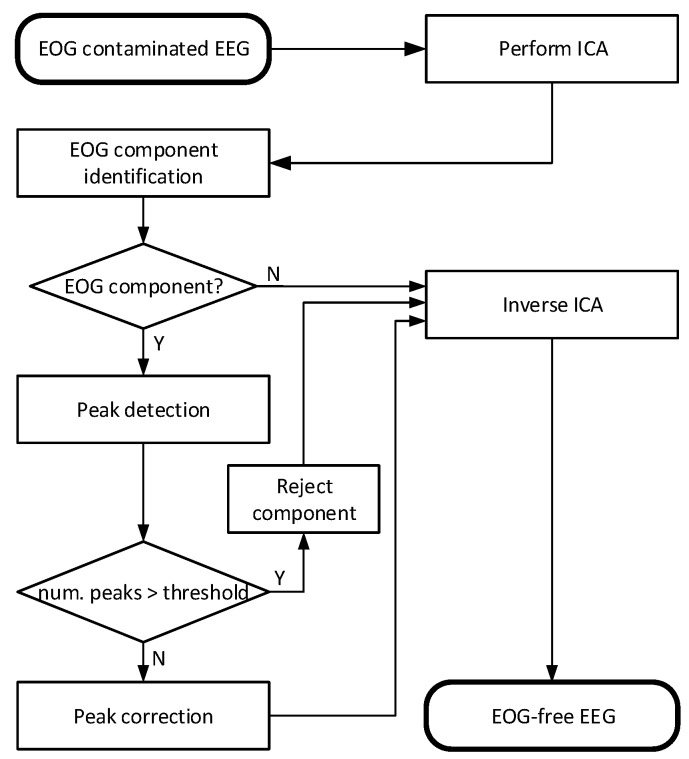
The data processing flowchart of the proposed EOG removal method.

In this section, the key steps of our proposed EOG artifact removal method are shown in algorithmic form and as a flowchart in Figure 1, followed by a detailed description of each step.

Algorithm: EOG removal:

Step 1 Each measured dataset is bandpass filtered (1–47 Hz, zero phase 4th order Butterworth), then re-referenced to the average reference.
Step 2 Infomax ICA is applied to the signal to estimate the source independent components.
Step 3 Automatic identification of the EOG component: the EOG component is identified based on the correlation between each component and data of each frontal EEG channel. The component with the highest correlation and above a threshold weight is selected as an EOG component.
Step 4 The identified EOG components are searched for EOG peaks.
Step 5 One-second windows are placed around the detected EOG peaks.
  (a) If the windows cover more than 60 percent of the given component, the entire component is marked for rejection. Continue at Step 7.
  (b) Otherwise, the EOG windows in the component are set as the target of artifact removal.
Step 6 Wavelet decomposition using Symlet *sym4* [17,39,40] wavelets of five levels is applied to decompose signals in each target window to different wavelet components, and only the high frequency components are retained for the signal reconstruction process. These retained components are used in the inverse wavelet transform to reconstruct the cleaned independent component.
Step 7 Using the inverse ICA process, the artifact free signals are estimated from the corrected components.

### 3.4. Method Details

**Figure 2:**
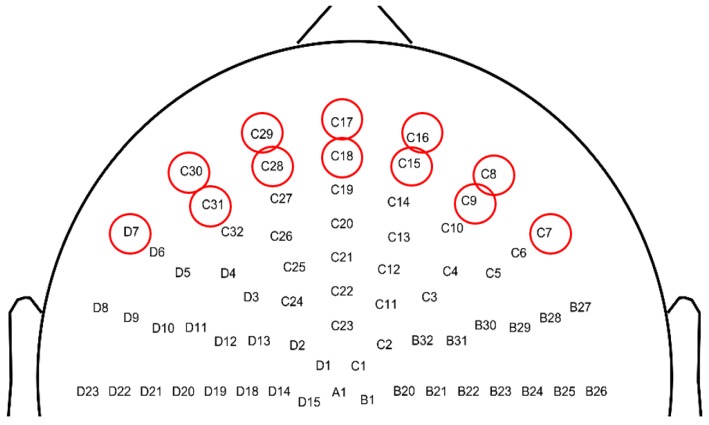
An example for frontal channels (marked by red circles) used for correlation calculation in EOG independent component identification. Top view of scalp with nose pointing upwards, 128-channel Biosemi ABC electrode layout.

In this section, the key steps of the proposed method were described in detail. Once the input signal is filtered and the ICA process was executed, the first key task was to identify which component represents EOG artifacts (Algorithm, Step 3). The Pearson correlation $R_{X,Y}$ between a given independent component Y and each of the frontal channels X (shown in Figure 2) was computed based on the underlying assumption that EOG artifacts appear primarily in the frontal channels and the component describing EOG activity should have high correlation with some of these channels. The Pearson-correlation between the frontal channels and the components was computed as

(4) $$R_{X,Y}=\frac{Cov\left(X,Y\right)}{\sigma_{X}\sigma_{Y}},$$

where $\sigma_{X}$ and $\sigma_{Y}$ are the standard deviations of channel *X* and component *Y*, respectively. Components with the highest R value are identified as candidate EOG components to be examined further. Naturally, a different set of frontal electrodes must be selected for different electrode layouts. The number of frontal channels does not affect the accuracy of the proposed method as long as there are at least two frontal channels on the forehead, one close to the left and one to the right eye. This ensures that high correlation between ocular artifacts and EOG components can be found.

**Figure 3:**
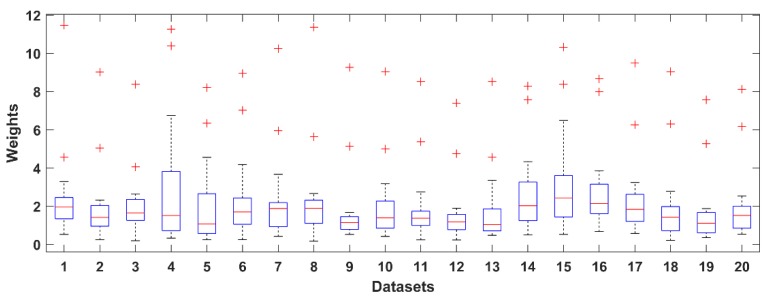
Distribution of the normalized weights of the components of 20 EOG contaminated measurements selected from the Klados datasets. The red crosses represent the weight of the EOG (HEOG and VEOG) components.

**Figure 4:**
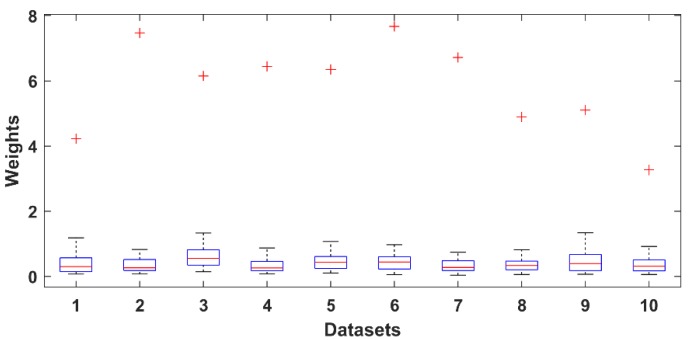
Distribution of the normalized independent component analysis (ICA) component weights of 10 selected PhysioNet datasets.

**Figure 5:**
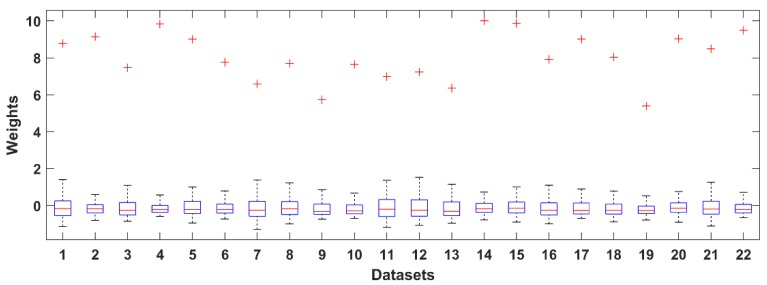
Distribution of the normalized ICA component weights of the 22 datasets obtained in our laboratory.

The candidate components were further examined for weight value distribution and only those with weights greater than a threshold were kept as EOG components. Elements of the weight vector **w** are defined as:

(5) $${\bar{w}}_{j}=\frac{1}{K}\overset{K}{\underset{i=1}{\sum }}|w_{ij}|,\;j=1,2,\ldots ,N,$$

where ${\bar{w}}_{j}$ is the average weight of component *j* over the frontal channels, $w_{ij}$ is the weight element of the mixing matrix **A**, *K* is the number of the frontal channels and *N* is the number of components. The distribution of values in the weight vectors was used to calculate a statistical threshold. The distributions are shown for all three datasets in Figure 3, Figure 4 and Figure 5 as boxplots. Red crosses represent weights for the EOG components. Note that the maximum value of each distribution acts as a reliable threshold for detecting the EOG component (outliers).

**Figure 6:**
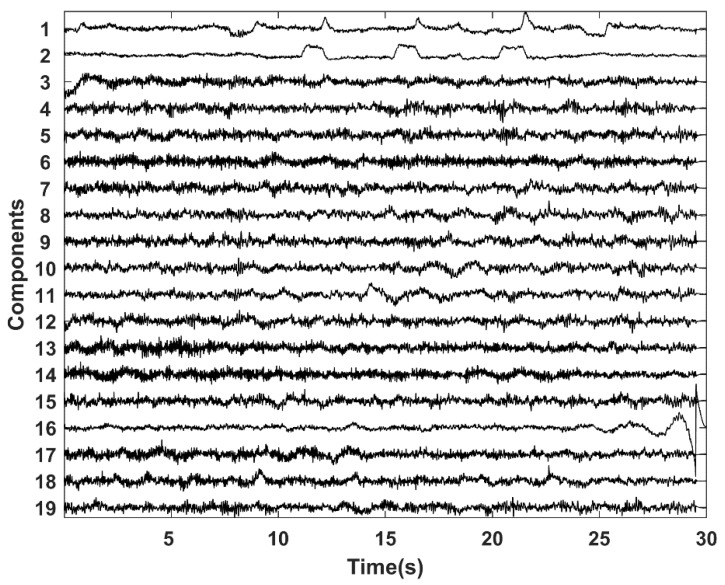
ICA components of a selected Klados dataset.

**Figure 7:**
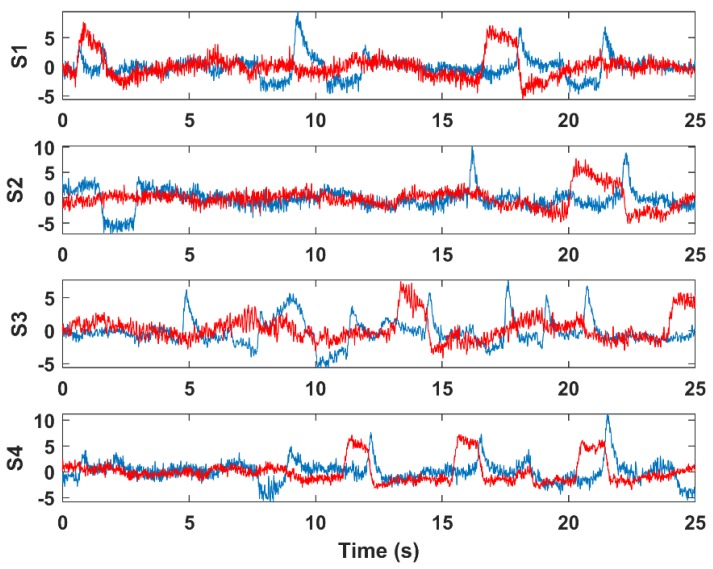
Sample sections of VEOG (blue) and HEOG (red) EOG components from four selected Klados datasets.

The threshold was computed from the distribution of weights and each weight vector element was tested against it to decide whether the component is in fact an EOG component:

(6) $$Y_{i}\;\mathrm{is}\;\mathrm{EOG},\;\mathrm{if}\;{\bar{w}}_{i}>Q_{3}\left(w\right)+1.5*IQR\left(w\right),\;i=1\ldots N,$$

where ${\bar{w}}_{i}$ is the weight of component *Y_i_*, **w** is the averaged weight vector and $Q_{3}$ and IQR are the upper quartile and interquartile range, respectively [41]. The result of this step is illustrated in Figure 6 and Figure 7. Figure 6 shows the independent components of a selected dataset. Components 1 and 2 contained EOG artifacts (blinks and eye movements, respectively). Figure 7 shows the result of the component selection and threshold application that identified the EOG components for the sample datasets 1–4.

The next step in the algorithm (Step 4) was the detection of EOG peaks within the components. First a normal peak detection was performed on the component values (finding local maxima [42]), then the peaks were further examined whether they were, in fact, EOG peaks. The decision whether a local maximum $m_{k}$ belongs to the set of EOG peaks *P* was made using the following rule containing amplitude and duration constraints.

(7) $$P=\{m_{k}\;|\;|Y_{i}\left(m_{k}\right)|>3\cdot E\left\{\left|Y_{i}\right|\right\}\;\mathrm{and}\;t\left(Y_{i}\left(m_{k}\right)\right)-t\left(Y_{i}\left(m_{k-1}\right)\right)\geq 0.5\;\sec\},$$

where $m_{k}$ is the *k*th peak in component $Y_{i}$ and $E\left\{\left|Y_{i}\right|\right\}$. is the expected value of the component vector $Y_{i}$ and t refers to the timestamp of peak $m_{k}$. Each two consecutive selected peaks must satisfy the peak amplitude condition and the between-peak time distance of 0.5 s to correctly classify peaks as EOG artifacts.

**Figure 8:**
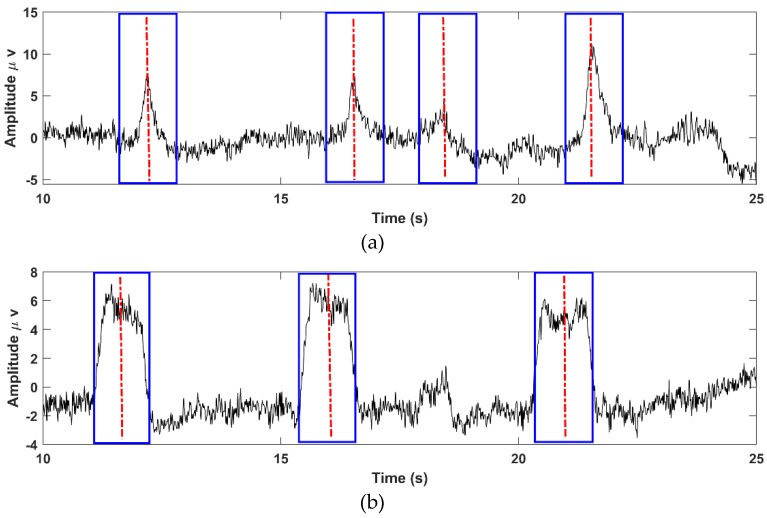
Correction target windows around the detected VEOG blink (**a**) and HEOG eye movement (**b**) peaks in the EOG ICA components.

After locating the EOG peaks, target windows were placed around the peaks for EOG artifact removal (Algorithm: Step 5). A window size of 1 s duration was used, as this spans the length of the EOG artifact waveforms [43,44]). These windows would equally designate vertical-EOG (VEOG) and horizontal-EOG (HEOG) sections. Figure 8 illustrates the results of this step showing the windows marking blink and eye movement EOGs, respectively.

Artifact removal was performed on the EOG components selectively, only within the target windows, using wavelet decomposition (Algorithm: Step 6). The discrete wavelet transform (DWT) of a signal *f*(*t*) is defined as

(8) $$F_{W}\left(j,k\right)=\frac{1}{\sqrt{2^{j}}}\sum_{t=0}^{N}f\left(t\right)\varphi \left(\frac{t-k2^{j}}{2^{j}}\right),$$

where φ is the wavelet basis function, *j* is the scale parameter and *k* is the shift parameter. The success of EOG detection in a component is dependent on the choice of wavelet basis function [17] and the level of decomposition [45]. Several wavelet basis functions, e.g., Haar, Daubechies, coiflet and Symlet, can be used to detect and correct EOG waveforms [39,46,47]. It has been shown [47] that the Symlet wavelet family (sym2 to sym20) is the most suitable for EOG peaks and has been used successfully in several artifact removal applications. The sym-4 wavelet was selected as final basis function due to its smallest error (RMSE) between the corrected and artifact-free signals [47]. Our tests with the Symlet wavelets confirmed the same results (mean RMSE—Haar: 9.85, db4: 7.42, sym3: 7.37, sym4: 6.29, sym5: 6.54, sym6: 6.96).

**Figure 9:**
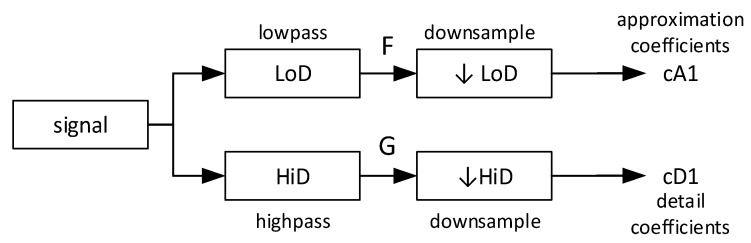
The wavelet decomposition process and calculation of coefficients. Letters F and G represent the output signals of the low-pass and high-pass filters, respectively.

The ICA component signal was decomposed into wavelet components by passing through a quadrature mirror filter performing low-pass and high-pass filtering followed by downsampling the input signal at each level of decomposition and generating the output coefficients related to lower and higher frequencies [48]. The details of this process are shown in Figure 9.

**Figure 10:**
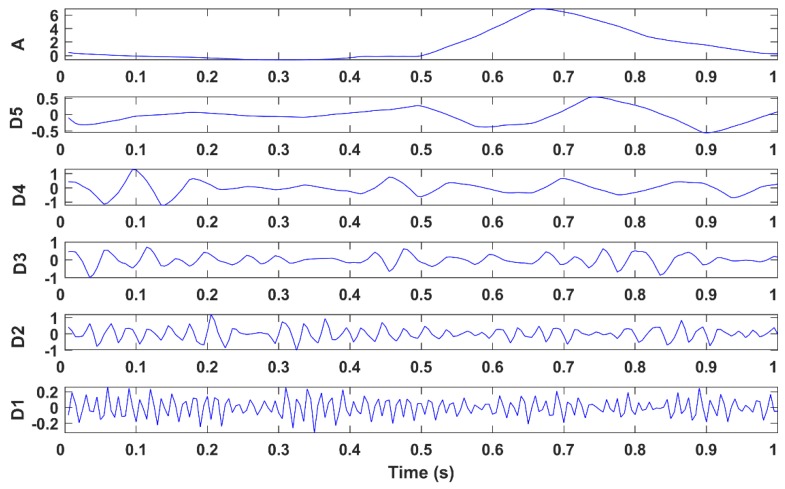
Wavelet decomposition of a target EOG peak signal window within an independent component.

Different levels of wavelet decomposition were tested to find the optimal parameters. Five levels of DWT were used to decompose the component into detail (D1:D5) and approximation coefficients (A), as illustrated in Figure 10. Coefficients D1:D3 represent the higher frequency components while coefficients D4:D5 while A represent low frequency components. Since the spectrum of the EOG artifacts is concentrated in the frequencies below 7 Hz [49], the signals were reconstructed only from coefficients D1:D3, which represent the high frequencies related to the EEG signal; the other components were discarded. The reconstructed signals were then projected back to the EOG components, and inverted to obtain the artifact free data. The proposed method was able to automatically detect and correct both the vertical EOG activity (blinks) and horizontal EOG artifacts (eyes movements), which made it suitable for unsupervised artifact removal applications.

### 3.5. Performance Metrics

The quality of artifact removal methods can be quantified by two basic types of metrics; metrics that describe the amount of artifact removed by a given cleaning method, and metrics that measure the distortion introduced in the signal by the cleaning process [50]. Two metrics of the first type are the artifact removal percentage λ and the signal-to-noise ratio difference [51]. When the true, uncontaminated EEG and the added artifact signals are known, the artifact removal percentage can be calculated as

(9) $$\lambda =100\left(1-\frac{R_{ref}-R_{cleaned}}{R_{ref}-R_{contam}}\right),$$

where $R_{ref}$ is the autocorrelation of the true EEG signal with time lag 1, $R_{cleaned}$ is correlation between the true EEG and the cleaned signals, while $R_{contam}$ is the correlation between the true EEG and the artifactual signals. When $R_{cleaned}$ is close to the reference $R_{ref}$, the negative term tends to 0, hence a high lambda value indicates high efficacy in artifact removal.

The difference in signal-to-noise ratio ΔSNR [51] is a similar measure characterizing the amount of artifact removed from the signals. It is defined as

(10) $$\Delta SNR=10{log}_{10}\left(\frac{\sigma_{x}^{2}}{\sigma_{e_{cleaned}}^{2}}\right)-10{log}_{10}\left(\frac{\sigma_{x}^{2}}{\sigma_{e_{contam}}^{2}}\right),$$

where $\sigma_{x}^{2}$ is the variance of the true EEG signal, and $\sigma_{e_{contam}}^{2}$ and $\sigma_{e_{cleaned}}^{2}$ are the variances of the error signals $e_{contam}\left(n\right)=r\left(n\right)-x\left(n\right)$ and $e_{cleaned}\left(n\right)=r^{\prime }\left(n\right)-x\left(n\right)$ with x(n), r(n) and $r^{\prime }\left(n\right)$ representing the true EEG, contaminated and the artifact cleaned signals, respectively.

Distortion in the time domain can be quantified using the root mean square error calculated between the true EEG x(n) and the cleaned signals $r^{\prime }\left(n\right)$.

(11) $$RMSE=\sqrt{\frac{1}{N}\sum_{n=1}^{N}{\left(r^{\prime }\left(n\right)-x\left(n\right)\right)}^{2}}.$$

Spectral distortion can be measured by the magnitude squared coherence (MSC) [52] that computes the frequency-domain correlation between the pure and the cleaned EEG signals:

(12) $$MSC=C_{xy}\left(f\right)=\frac{{\left|R_{xy}\left(f\right)\right|}^{2}}{R_{xx}\left(f\right)R_{yy}\left(f\right)}\;,$$

where $R_{xy}\left(f\right)$ is the cross spectral density between the two signals *x* and *y* at frequency f, and $R_{xx}\left(f\right)\;\mathrm{and}\;R_{yy}\left(f\right)$ are the autospectral density of *x* and *y*, respectively. MSC is a frequently used metric for evaluating frequency-related distortions after artifact removal [6,53,54,55,56,57].

## 4. Results

This section presents the performance evaluation of the proposed EOG removal method. Three datasets mentioned in Section 3 were used; the Klados, the PhysioNet and the laboratory resting-state datasets. For each dataset, the proposed method (PM) is compared to the traditional full component rejection method (ICArej) [58] and the wavelet-enhanced ICA (wICA) [6] component correction methods using the performance metrics specified in Section 3.4. wICA is also compared to rejection ICA to confirm its claimed higher performance.

### 4.1. Semi-Simulated EEG Dataset

**Figure 11:**
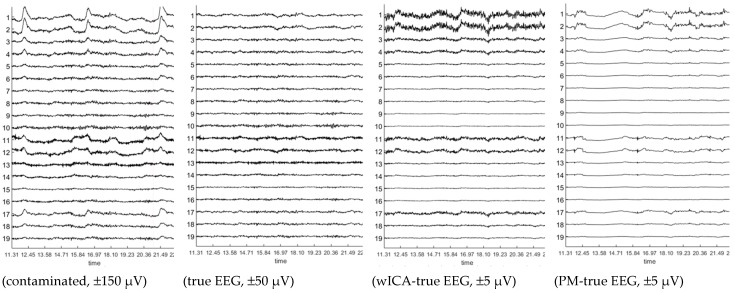
Illustration of the cleaning performance on one artifact contaminated section of the Klados dataset9. The two subplots on the right show the difference of the pure electroencephalography (EEG) data and the wavelet-enhanced ICA (wICA) and proposed method (PM) cleaned signals, respectively. Amplitude scales are different to make difference signal visible.

**Figure 12:**
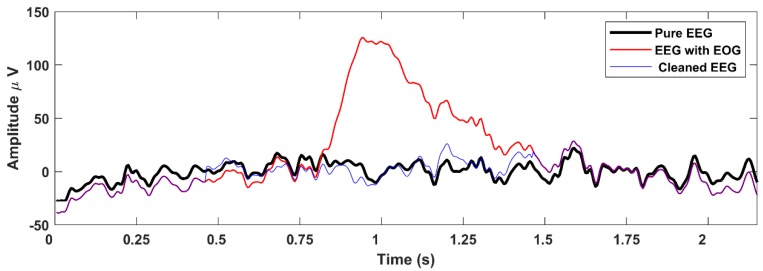
Comparison of the artifact-free, the contaminated and the PM-cleaned EEG signals of dataset9, channel Fp1.

The performance of the proposed method was first evaluated on the Klados datasets [36]. These measurements contain semi-simulated signals, containing resting-state measured signals with and without added simulated EOG contamination. Access to the pure EEG signal allows for calculating accurate performance metrics. For illustrative purposes, Figure 11 shows the contaminated and pure EEG signals, as well as the absolute difference between the wICA-cleaned signal and the pure EEG, and the difference of the signal cleaned with the proposed method and the pure EEG signal. Note that the amplitude scales are different in order to make the difference signals visible. The contaminated segment shows three strong blink (ch 1–4, 17–19) and two eye movement (ch 11–12) artifacts. Note the difference between the difference signals (wICA–EEG_true_, PM–EEG_true_) obtained after cleaning with the wICA and the proposed method. The high-frequency content in the wICA difference signal indicates the removal of non-EOG signal components. Figure 12 shows a zoomed-in section of dataset12 (channel Fp1) illustrating how the PM cleaning method leaves the EEG signal intact outside the EOG zones, and how it follows the true EEG within the zones. The figures qualitatively indicate the improved removal quality of the proposed method.

**Figure 13:**
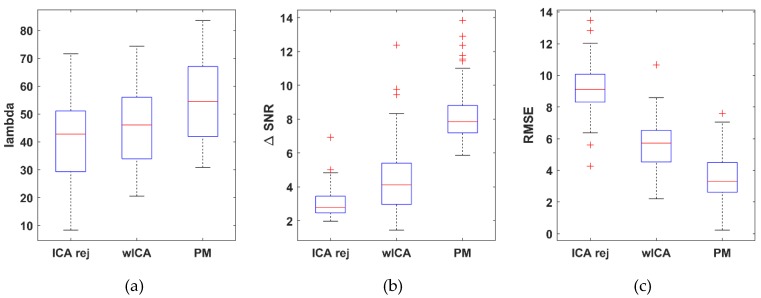
Distribution of the λ (**a**), difference in signal-to-noise ratio ΔSNR (**b**) and root mean square error RMSE (**c**) dataset average values obtained with the rejection ICA, wICA and the proposed method. For λ and ΔSNR the higher, while for RMSE, the lower values mean better performance.

A quantitative statistical comparison was performed on the entire dataset (54 measurements), in which the λ, ΔSNR, RMSE and MSC metrics were computed for each channel in each dataset with the three removal methods (rejection ICA, wICA and the proposed method) under study. After the performing channel, the distributions of the metrics for the dataset population are shown in Figure 13. Each metric value set was checked for normality and equal variance (F-test). A two-sample *t*-test (α = 0.05) was performed to decide whether there is a significant difference in performance between the PM and the wICA/ICArej methods for any metric. Performance of the wICA with respect to the rejection ICA method was also examined to verify claims that wICA outperforms rejection-based removal.

The λ value showed no significant difference (average improvement: 11.34%, *p* = 0.102) between the wICA and the reject ICA methods. The proposed method on the other hand was significantly better (19.1%, *p* = 0.00236) than the wICA and 32.6% better (*p* = 1.43 × 10^−5^) than the reject ICA methods. With respect to the ΔSNR metric, the wICA method was significantly better than the reject ICA method (50.05%, *p* = 2.08 × 10^−5^). The proposed method, however, resulted in significantly increased SNR compared to wICA (79.5%, *p* = 7.78 × 10^−15^) and reject ICA better (169.34%, *p* = 7.96 × 10^−36^). The RMSE results were similarly positive; wICA improved upon reject ICA by 39.1% (*p* = 3.89 × 10^−18^), while the proposed method showed 36.32% improvement (*p* = 5.84 × 10^−33^) over the wICA and 61.22% over the reject ICA (*p* = 5.80 × 10^−9^) methods, reducing the average RMSE from 5.579 (wICA) to 3.553 μV.

In addition to the statistical analysis, for enabling side-by-side comparison with the wICA method, Table 1 lists the RMSE values for the exact same datasets and channels that were reported in [6].

**Figure 14:**
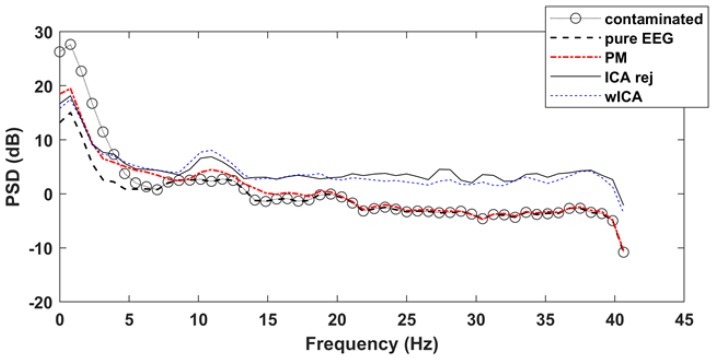
Power spectral density distributions of the pure, contaminated versus the ICA rej, wICA and PM method cleaned signals (dataset12, channel Fp1).

While the RMSE result indicates improved removal quality in the time domain, a key question remained as to how the spectral characteristics of the signal change after cleaning. Figure 14 illustrates the effect of artifact removal on the power spectral density of the EEG signals. The frontal channel Fp1 of dataset12 was used to show the difference among the difference methods. Note how the contaminated signal introduces strong δ−θ frequency band distortions. The reject ICA and wICA methods decreased this low frequency distortion but introduced higher α and β band frequency power increase. The proposed method, on the other hand, removed low frequency artifact-related distortions and followed the power density distribution of the pure EEG signal for higher frequencies with very little error.

**Figure 15:**
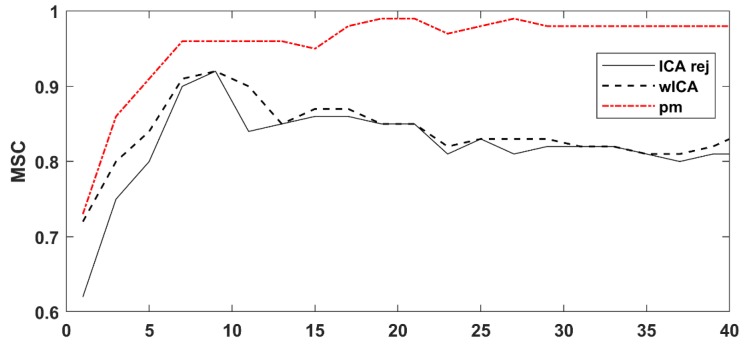
The grand average (20 datasets) magnitude squared coherence (MSC) results of the three cleaning methods. Note the higher average performance of our proposed method.

Performing the analysis for the entire dataset, the magnitude squared coherence after cleaning with the proposed method was 13.69% better (*p* = 4.20 × 10^−8^) than the wICA results and 15.93% better (*p* = 3.91 × 10^−8^) than the reject ICA values. No significant difference was found between the wICA and reject ICA results (*p* = 0.335). Figure 15 shows the overall grand average MSC results for the three methods. The performance advantage of our proposed method over the rejection ICA and wICA methods was clearly demonstrated.

**Figure 16:**
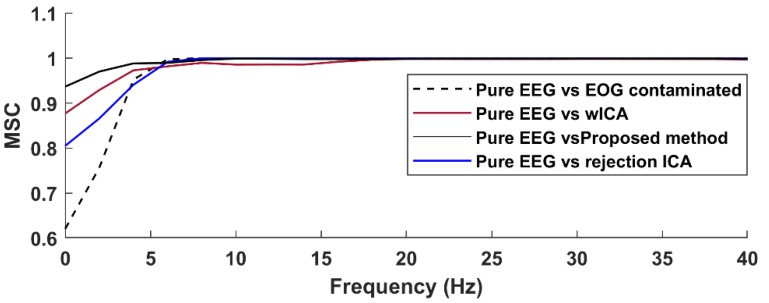
The magnitude squared coherence (MSC) between the pure EEG signal and the contaminated signal as well as the various cleaned signals (dataset12, Fp1).

Figure 16 shows, for a selected single frontal channel (Fp1, dataset12), the magnitude squared coherence in order to compare the spectral accuracy of the different EOG removal methods in a non-averaged manner. The results indicate that the different EOG artifact cleaning methods produced different spectral distortion in frequencies below 7 Hz. Coherence was the lowest for the uncleaned, EOG contaminated signal. The rejection-based ICA and wICA methods both reduced this distortion, but it was our proposed method that produced coherence values closest to the ideal value of 1. Note that wICA also introduced a slight distortion in the 7–17 Hz range as well, which might be the result of unnecessary removal of higher frequency wavelet components.

### 4.2. Resting State EEG Dataset

**Figure 17:**
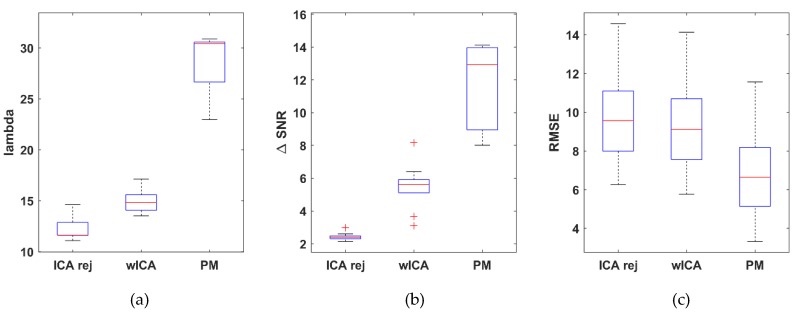
Distribution of the λ (**a**), ΔSNR (**b**) and RMSE (**c**) dataset average values for the resting state laboratory measurements obtained by cleaning with the rejection ICA, wICA and PM methods. For λ and ΔSNR the higher, while for RMSE, the lower values mean better performance.

To evaluate the performance of our method on real EEG data, 2–3 min long 128-channel resting state EEG measurements of 10 subjects (obtained in our laboratory) were used. Since the true, artifact-free EEG signals are unknown in this case, modified performance metrics were used. The true EEG signal was estimated for each subject from a manually selected 5-s long artifact-free segment. The datasets were then cleaned with the three different methods, and partitioned into 5-s long segments. The performance metrics were subsequently calculated by using the entire signal (all 5-s segments) with respect to the reference segment in the corresponding formulae. The distribution of the results for each method is shown in Figure 17. While the range of values are lower (λ and ΔSNR) or higher (RMSE) than those obtained for the semi-simulated Klados dataset (due to the different estimation of the true EEG), the trend in performance was the same.

Performing the same statistical analysis as for the semi-simulated Klados datasets, the proposed method achieved 154.61% (*p* = 6.86 × 10^−9^) improvement for λ over the wICA and 136.88% better (*p* = 8.28 × 10^−10^) than the reject ICA methods. The wICA method achieved 6.97% (*p* = 2.06 × 10^−5^) improvement over the reject ICA method. With respect to the ΔSNR metric, the proposed method achieved 388.88% improvement (*p* = 7.83 × 10^−7^) over the reject ICA and 116.45% (*p* = 6.28 × 10^−6^) over the wICA method. The wICA method performed better than reject ICA by 125.87% (*p* = 5.80 × 10^−5^). The RMSE results showed the proposed method achieved 26.94% improvement (*p* = 0.039) over the wICA and 30.37% over the reject ICA (*p* = 0.0165) methods. No significant difference was found between the wICA and reject ICA methods (4.7%, *p* = 0.6887). For the spectral coherence MSC, the proposed method improved over both the wICA (19.12%, *p* = 5.89 × 10^−5^) and the reject ICA (23.5%, *p* = 6.73 × 10^−6^) methods. On the other hand, no significant difference was found between the reject ICA and wICA methods (3.68%, *p* = 0.423479).

**Figure 18:**
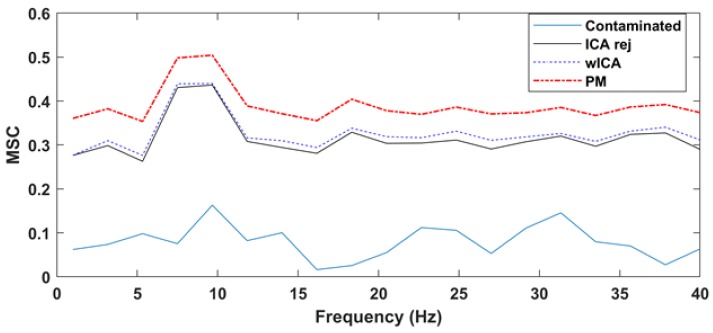
MSC values obtained with different cleaning methods for the resting state laboratory dataset (20 subjects).

Similar results were obtained for the spectral distortion. As shown in Figure 18, the Magnitude Squared Coherence (MSC) values for the proposed method were significantly higher than for the ICArej and wICA methods.

**Figure 19:**
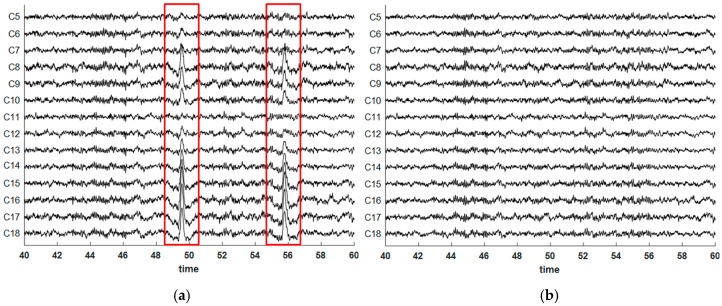
A 128-channel EOG contaminated EEG dataset before (**a**) and after (**b**) artifact removal.

**Figure 20:**
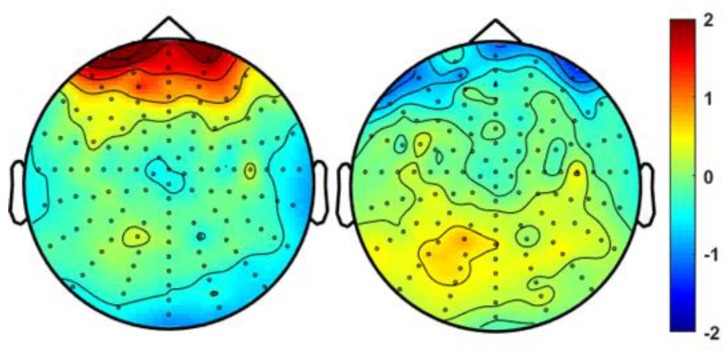
Topoplot potential map (µV) of a 128-channel EOG contaminated resting state measurement before (left) and after artifact removal (right).

As a qualitative illustration of the effect of our removal method on real measurement data, Figure 19 shows a 20-s section of the contaminated resting state EEG before and after EOG removal (proposed method). Figure 20 illustrates the same effect on a 2D scalp potential map at the peak of an EOG artifact. The EOG artifact was clearly visible in the frontal area that disappeared after cleaning. Note also the emerging parietal topography in the cleaned version, which was almost completely hidden in the contaminated map.

### 4.3. PhysioNet P300 ERP Dataset

#### 4.3.1. Peak Detection Performance

The accuracy of peak detection is crucial in the proposed method. Since the Klados and PhysioNet datasets contain annotations for EOG events, these were used to verify the performance of our EOG peak detection approach. Peak detection performance was characterized by the sensitivity measure, *Se* = TP/(TP + FN), where TP is the number of true positive (accurately detected) and FN is the number of false negative (missed) peaks. The results were as follows. Klados dataset (218 EOG peaks, TP = 217, FN = 1): Se = 99.54% and PhysioNet dataset (78 EOG peaks, TP = 78, FN = 0): Se = 100%.

#### 4.3.2. Artifact Removal Performance

The PhysioNet P300 dataset was originally created to detect and classify P300 peaks in the BCI speller experiment [37,38] and as such, can be used to examine the proposed method for cleaning task-oriented event related potential data. Two tests were conducted to verify whether or not the cleaning methods distort ERP waveforms and peaks. First, a statistical analysis was performed on the RMSE values to verify the presence of significant improvements; second, the distortion effects of the different cleaning methods were examined.

**Figure 21:**
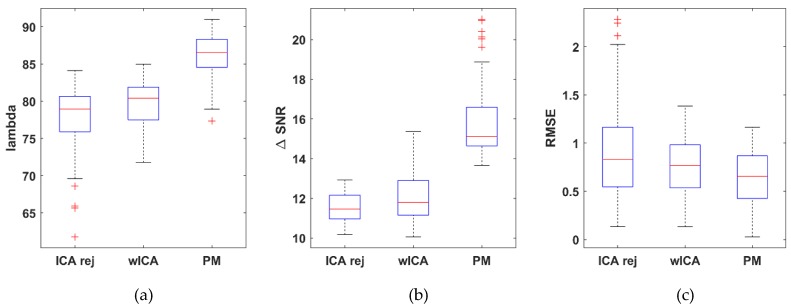
Distribution of the λ (**a**), ΔSNR (**b**) and RMSE (**c**) dataset average values for the PhysioNEt P300 dataset by cleaning with the rejection ICA, wICA and PM methods. For λ and ΔSNR the higher, while for RMSE, the lower values mean better performance.

For the statistical analysis, from among the target and non-target epochs, the target epochs were selected that elicit the P300 component. These resulted in 21 stimulus-locked epochs of length 500 ms extracted from the original contaminated 64-channel measurements for each subject (subjects s03, s04, s08 and recording *rc02*). From these 21 epochs, the artifact free epochs were selected and averaged for estimating the reference, pure P300 ERP signal $ERP_{ref}$ (number of epochs varied from 16 to 19) and averaged to generate a pure reference ERP signal. The contaminated P300 ($ERP_{contam}$) was computed by averaging the 21 uncleaned epochs. Then, the original recordings were cleaned with the three removal methods in question (rejection ICA, wICA and PM), and an ERP signal for each method was generated by averaging the 21 segments of the cleaned signals resulting in $ERP_{clean}^{rej}$, $ERP_{clean}^{wICA}$ and $ERP_{clean}^{PM}$. Since the ERP waveforms differ from channel to channel, the channels were not averaged to calculate group statistics. Instead, subjects were selected individually then a statistical test was performed using the 64 channel-ERPs as sample population for pairwise comparison or the removal methods. The two-sample *t*-tests for each subject produced the results shown in Table 2. The proposed method performed significantly better than the wICA or rej ICA methods for each subject. Similar outcome is obtained for the *λ* and ΔSNR performance metrics. The distribution of the results for each method is shown in Figure 21.

**Figure 22:**
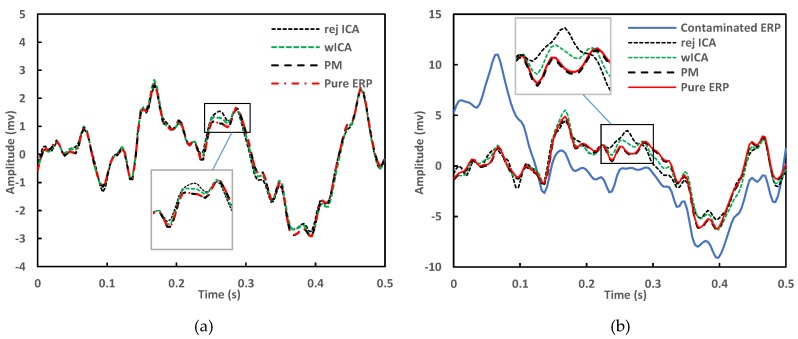
Event related potential (ERP) signals computed from artifact-free epochs only (**a**) and ERP signals computed from all cleaned epochs (**b**) showing the distorting effects of the cleaning methods on ERP curves. $ERP_{clean}^{PM}$ produced the smallest difference in both cases (dataset, electrode Fpz).

The distortion of the removal methods was tested by two ways. First, the pure ERP signal was compared to the cleaned ERP signals averaged from the same epochs as the pure ERP (artifactual epochs excluded). This shows the distortion of each method operating on artifact-free data (Figure 22). The rej ICA and wICA introduce larger distortions, since the entire signal is affected by EOG removal, even if only artifact-free epochs are averaged afterwards. By using the proposed method, however, artifact-free sections of the signal are unaffected and the averaged clean epochs are nearly identical to the reference signal. See inset in Figure 22a.

In the second test, all epochs of the cleaned signals were used to compute the ERP signal and compared to the pure reference ERP. This shows the ability of the method to recreate the pure ERP waveforms after artifact removal. Figure 22b shows the ERP distortion under real conditions, in presence of EOG artifacts. The solid blue line indicates the contaminated P300 signal. The proposed method follows the pure ERP curve with the smallest error, see inset in Figure 22b.

## 5. Discussion

EOG artifacts are random, high-amplitude distortions in EEG recordings that, if appear frequently, can make entire measurements unusable. Due to the unpredictable nature of artifacts, traditional artifact removal is based on manually data inspection and rejection of contaminated data segments. This process is both time-consuming and prone to human errors. The introduction of independent component analysis for artifact removal [11] revolutionized the field, first by providing a theoretical framework for separating artifacts, then secondly, by paving the way to automatic, intervention-free implementations. Unfortunately, the strong statistical independence assumption of ICA does not always hold in practice, resulting in neural data leaking into artifact components. In these cases, independent component rejection based artifact removal methods lose valuable neural activity information.

The wavelet-enhanced ICA (wICA) [6] method showed that independent components can be cleaned from artifacts if they are not rejected entirely. As shown in the Results section, correcting components this way not only preserves information, but also reduces distortions that rejection ICA methods introduce in the time and frequency domain. Distortions in the frequency domain, for instance, can corrupt EEG-based connectivity analyses [6].

The novelty of the method proposed in this paper was that component artifact correction was only performed in EOG contaminated sections of the component, ensuring that non-EOG contaminated sections were left untouched. The statistical analysis of the artifact removal performance metrics confirmed that while wICA outperformed rejection ICA methods in most performance parameters our proposed method significantly outperformed the quality of both the wICA and the rejection ICA EOG cleaning methods, both in the time and spectral domains, resulting in close-to-ideal pure EEG signals.

## 6. Conclusions

This paper described an improved wavelet-based ICA method for removing EOG components from EEG measurements. The method operated on independent components produced by an independent component analysis, automatically selected EOG-contaminated components for subsequent wavelet decomposition. EOG peaks were detected in the selected components, then the wavelet components representing EOG artifact waveforms were removed in windows placed around the EOG peaks. The component was then reconstructed from the remaining wavelet coefficients and used in signal reconstruction using the inverse ICA process. This partial component cleaning approach significantly outperformed the popular wICA and rejection ICA based artifact removal methods in all key artifact removal performance metrics. In addition, our method was fully automatic; it did not require manual component and artifact inspection, which could simplify and speed up high-quality artifact removal processes. Our future research will focus on implementing this method using high-performance computing techniques to support very fast and potentially online artifact cleaning.

## Figures and Tables

**Table 1:** RMSE values of the different artifact removal methods for the Klados dataset.

Dataset, Channel	Contaminated EEG		Cleaned EEG	
		Rejection ICA	wICA	Proposed Method
Dataset 1, FP1	34.9	16.3	12.6	7.9
Dataset 1, F8	13.7	9.4	7.3	3.2
Dataset 2, FP1	37.8	14.6	8.7	4.6
Dataset 2, F8	15.9	8.4	5.4	1.5
Dataset 9, FP1	30.8	18.9	9.2	3.2
Dataset 9, F8	15.5	12.7	6.4	2.6
Dataset 12, FP1	38.4	14.9	9.8	7.2
Dataset 12, F8	18.8	11.3	7.2	3.5

**Table 2:** RMSE improvement between methods. Bold values mark significant differences.

Dataset	RMSE Improvement (%)		
	PM vs. rej ICA	PM vs. wICA	wICA vs. rej ICA
s03, rc02	**17.16** (*p* = 4.74 × 10^−4^)	**34.18** (*p* = 0.0286)	**20.55** (*p* = 0.049)
s04, rc02	**24.64** (*p* = 0.0018)	**16.46** (*p* = 0.0264)	9.79 (*p* = 0.2062)
s08, rc02	**25.62** (*p* = 0012)	**14.43** (*p* = 0.0348)	13.08 (*p* = 0.0992)
